# Relationship Between Adherence to Remote Monitoring and Patient Characteristics: Observational Study in Women With Pregnancy-Induced Hypertension

**DOI:** 10.2196/12574

**Published:** 2019-08-28

**Authors:** Thijs Vandenberk, Dorien Lanssens, Valerie Storms, Inge M Thijs, Lotte Bamelis, Lars Grieten, Wilfried Gyselaers, Eileen Tang, Patrick Luyten

**Affiliations:** 1 Faculty of Medicine and Life Sciences Hasselt University Diepenbeek Belgium; 2 Department of Obstetrics & Gynecology Ziekenhuis Oost-Limburg Genk Belgium; 3 Future Health Ziekenhuis Oost-Limburg Genk Belgium; 4 Limburg Clinical Research Center Hasselt University Diepenbeek Belgium; 5 Mobile Health Unit Hasselt University Diepenbeek Belgium; 6 Centre for Translational Psychological Research TRACE Ziekenhuis Oost-Limburg Genk Belgium; 7 Faculty of Psychology and Educational Sciences Katholieke Universiteit Leuven Leuven Belgium; 8 Research Department of Clinical, Educational and Health Psychology University College London London United Kingdom

**Keywords:** remote monitoring, gestational hypertensive diseases, monitoring, ambulatory, hypertension, pregnancy-induced, surveys and questionnaires, treatment adherence and compliance

## Abstract

**Background:**

Pregnancy-induced hypertension (PIH) is associated with high levels of morbidity and mortality in mothers, fetuses, and newborns. New technologies, such as remote monitoring (RM), were introduced in 2015 into the care of patients at risk of PIH in Ziekenhuis Oost-Limburg (Genk, Belgium) to improve both maternal and neonatal outcomes. In developing new strategies for obstetric care in pregnant women, including RM, it is important to understand the psychosocial characteristics associated with adherence to RM to optimize care.

**Objective:**

The aim of this study was to explore the role of patients’ psychosocial characteristics (severity of depression or anxiety, cognitive factors, attachment styles, and personality traits) in their adherence to RM.

**Methods:**

Questionnaires were sent by email to 108 mothers the day after they entered an RM program for pregnant women at risk of PIH. The Generalized Anxiety Disorder Assessment-7 and Patient Health Questionnaire-9 (PHQ-9) were used to assess anxiety and the severity of depression, respectively; an adaptation of the Pain Catastrophizing Scale was used to assess cognitive factors; and attachment and personality were measured with the Experiences in Close Relationships-Revised Scale (ECR-R), the Depressive Experiences Questionnaire, and the Multidimensional Perfectionism Scale, respectively.

**Results:**

The moderate adherence group showed significantly higher levels of anxiety and depression, negative cognitions, and insecure attachment styles, especially compared with the over adherence group. The low adherence group scored significantly higher than the other groups on other-oriented perfectionism. There were no significant differences between the good and over adherence groups. Single linear regression showed that the answers on the PHQ-9 and ECR-R questionnaires were significantly related to the adherence rate.

**Conclusions:**

This study demonstrates the relationships between adherence to RM and patient characteristics in women at risk of PIH. Alertness toward the group of women who show less than optimal adherence is essential. These findings call for further research on the management of PIH and the importance of individual tailoring of RM in this patient group.

**Trial Registration:**

ClinicalTrials.gov NCT03509272; https://clinicaltrials.gov/ct2/show/NCT03509272

## Introduction

Pregnancy-induced hypertension (PIH), which is a complication in 6% to 10% of pregnancies, is defined as a systolic blood pressure (BP)>140 mmHg and diastolic BP>90 mmHg. PIH refers to 1 of 4 conditions: (1) pre-existing hypertension, (2) gestational hypertension, (3) pre-eclampsia, and (4) unclassifiable hypertension [1]. It is a major cause of maternal, fetal, and neonatal morbidity and mortality [1,2]. The assessment of women with pregnancies complicated by PIH includes clinical follow-up, serological investigations, and fetal ultrasound. The type and frequency of follow-up depends on the kind and severity of the hypertensive disorder [1]. The goal of treatment is to prevent significant cerebrovascular and cardiovascular events in the mother, without compromising fetal well-being [3].

New techniques to support these strategies have recently been developed, including remote monitoring (RM), which can be broadly defined as the use of telecommunication technologies to facilitate the transmission of medical information and services between health care providers and patients [4]. RM is a relatively new approach (dating back to the early 1990s) that facilitates patient management at home [5]. As part of the Hasselt University and Limburg Clinical Research Program, Ziekenhuis Oost-Limburg (ZOL, Genk, Belgium), a large hospital in the east of Belgium, added RM to the prenatal care of women with PIH. All women diagnosed with PIH who delivered at the outpatient prenatal clinic of ZOL were included. Women received RM on demand of the responsible obstetrician before admission or after discharge from the prenatal ward. The criteria to initiate RM were PIH at gestational age more than 12 weeks where an intensive follow-up until delivery was desirable. Women without a mobile phone, a gestational age less than 12 weeks, a fetus with congenital malformations, and women who refused informed consent were excluded and received conventional care. Women consenting for RM were asked to perform 2 BP measurements a day with the iHealth Blood Pressure Monitor (iHealth Feel), fill in once a week their weight on the app, and to wear continuously an iHealth activity tracker (iHealth Wave; iHealth, Paris, France). The data from the monitor devices were transmitted to a Web-based dashboard developed by the Mobile Health Unit of the University of Hasselt and the Future Health Department of ZOL. Predetermed alarm signals were set; 1 midwife performed remote follow-up of all transformed data at the dashboard. Alarm events were communicated with the obstetrician in charge to discuss management options before contacting and instructing patients at home. Therapeutic interventions were according to local management. Our first results were promising, suggesting that the addition of RM to the prenatal care protocol for women at risk of gestational hypertensive disease reduces prenatal hospitalization (until the moment of delivery), inductions, and pre-eclampsia compared with the levels in women who receive conventional care. Furthermore, it is likely that women monitored with RM will enter labor spontaneously and will be more likely to be diagnosed with gestational hypertension rather than pre-eclampsia than women treated with conventional care [6]. RM has also been effective in the follow-up of pregnant women with issues such as problematic BP and bodyweight [7,8]. However, adherence to RM is an important concern. Several studies have reported low rates of adherence to RM [9-11]. Adherence refers to the extent to which a patient follows a prespecified treatment regimen or protocol [12]. The methods used to measure treatment adherence are either direct or indirect. Direct methods include observation and the assessment of metabolites or biological markers in the blood. Indirect methods include self-report questionnaires, pill counts, rate of prescription refilling, and the clinical assessment of patients’ physiological markers [13,14].

In developing new strategies (including RM) to optimize the obstetric care of pregnant women, it is important to investigate the patients’ characteristics, as these will potentially affect their adherence to RM. The peripartum period has long been known to be associated with increased levels of stress and anxiety, related to the transition to parenthood and parental tasks and concerns associated with this transition [15]. However, when PIH is present, it potentially increases the already elevated levels of stress and anxiety associated with this normative and normally adaptive heightened “maternal preoccupation” in the perinatal period [16,17]. In this context, cognitive factors, such as catastrophizing or rumination, and insecure attachment styles and personality factors, such as perfectionism and dependency, have been associated with problems in negotiating the challenges of parenthood, which are expressed as increased levels of anxiety and depression [18-20]. Mothers with a tendency to catastrophize, for instance, might be excessively worried about PIH and might, therefore, show either poor adherence to RM because they wish to avoid potentially threatening information or alternatively, they might engage excessively in RM, and RM might become an obsession for them. Individuals with avoidant attachment styles and high levels of self-critical perfectionism might show a similar pattern of avoidance or over adherence, whereas those with anxious attachment or dependent personality traits might become overly compliant with RM. Poor medical outcomes and higher mortality rates in individuals with attachment avoidance and self-critical perfectionism have been associated with a tendency to deny health problems and compulsive autonomy (the belief that one must be able to manage one’s problems on one’s own) [21]. In contrast, individuals with high levels of attachment anxiety and dependency traits typically seek help readily, typically leading to better health outcomes (eg, earlier detection of cancer), but also with excessive use of medical care [22-24]. Regarding perfectionism, different dimensions have been discerned [25]: self-oriented perfectionism refers to having high personal standards and the need to constantly live up to these high standards, whereas other-oriented perfectionism refers to expecting perfection and high performance from others; finally, socially prescribed perfectionism refers to a constant striving to live up to others’ high standards and expectations. Individuals with high levels of self-oriented or socially prescribed perfectionism might show excessive adherence to RM, whereas individuals with increased levels of other-oriented perfectionism might show low adherence because of a skeptical attitude toward others and the RM program in particular.

To the best of our knowledge, no research to date has examined the relationships between adherence to RM and patient characteristics. Therefore, the primary endpoint of this study was to explore the roles of depression and anxiety, cognitive factors, and attachment and personality traits in relation to adherence to RM. On the basis of the findings discussed above, we expected that anxiety and depression, and cognitive factors, such as rumination and catastrophizing, would be increased in low and excessively adherent mothers. Similarly, we expected high levels of attachment avoidance and self-critical perfectionism to be associated with low or over adherence. Furthermore, we hypothesized high levels of attachment anxiety and dependent personality features to be related to over adherence. Finally, we expected high levels of self-oriented or socially prescribed perfectionism to be associated with over adherence, whereas high levels of other-oriented perfectionism were hypothesized to be related to low adherence. The secondary endpoint of the study was the relation between the individual questionnaire and the adherence rate.

## Methods

### Study Protocol

This study is part of the Pregnancy Remote Monitoring (PREMOM) study, an observational study involving 8 hospitals in Limburg (Belgium), undertaken to optimize gestational outcomes in pregnancies complicated with PIH. The PREMOM protocol and main results have been reported elsewhere [6,26,27]. Briefly, women consenting to RM underwent obstetric surveillance with a wireless BP monitor and an activity tracker. They were asked to make 1 BP measurement in the morning and 1 in the evening, to enter 1 weight measurement weekly and to wear the activity tracker 24 hours a day until delivery or hospital admission. When alarm signals were detected (systolic BP>140 mmHg, diastolic BP>90 mmHg, or weight gain>1 kg/day) by the responsible midwife, the obstetrician-in-charge was contacted to discuss the management options before the patient was contacted at home. The types of interventions were (1) expectant management, (2) ambulatory blood sampling and 24-hour urine collection at home, (3) adjustment of antihypertensive therapy and/or physical activity, (4) admission to the prenatal ward, or (5) induction of labor. The therapeutic interventions were based on local management strategies.

Pregnant women were given information about the study at the start of their RM program. All the women provided written informed consent to participate in the study. The Ziekenhuis Oost-Limburg Medical Ethics Committee approved the study.

The characteristics of the participants were collected at inclusion in the PREMOM program. Demographic and obstetric information was collected at recruitment and after delivery from the hospital administration and/or billing records.

All participants received an email containing a SurveyMonkey link. After logging in, the participants were asked to complete 6 questionnaires (see subsection Questionnaires).

### Participants

A total of 124 mothers from the PREMOM study were invited to participate in this study. A total of 7 (5.65%) of them declined participation because of lack of interest. Of the remaining 117 pregnant women, 7 (5.98%) were hospitalized in the prenatal ward with complications before they could complete the questionnaires. In total, 110 pregnant women (88.71%) completed the questionnaires, 2 of whom were excluded from the final analysis because their data were invalid because of failure to fill out the questionnaires correctly.

### Questionnaires

The Generalized Anxiety Disorder-7 (GAD-7) assessment scale was used to assess anxiety. It consists of 7 items to be rated on a 4-point Likert scale ranging from 0 to 3. The Patient Health Questionnaire-9 (PHQ-9) was used to measure the severity of depression. This questionnaire consists of 9 items to be rated on a 4-point Likert scale ranging from 0 to 3. The Pain Catastrophizing Scale assesses painful experiences and indicators of negative thoughts. It consists of 13 items to be scored on a balanced 5-point Likert scale ranging from 1 to 5. This questionnaire was adapted by the research team to include pregnancy-related questions. Anxious and avoidant attachment styles were measured by the 36 items from the Experiences in Close Relationships-Revised Scale (ECR-R), to be rated on a 7-point Likert scale ranging from 1 to 7. The Depressive Experiences Questionnaire for Adolescents was used to assess self-criticism and dependency, consisting of 20 items to be scored on a 7-point Likert scale ranging from 1 to 7. Finally, the Multidimensional Perfectionism Scale measures self-oriented, other-oriented, and socially prescribed dimensions of perfectionism, using 45 items to be rated on a balanced 7-point Likert scale ranging from 1 to 7. For all 6 questionnaires, higher scores indicate higher levels of anxiety, depression, cognitive, attachment, or personality traits of interest.

### Adherence

Patients’ adherence to their scheduled daily measurements was determined by tracking the total number of scheduled events and then counting the actual number of measurements made. This from the moment of inclusion, until 90 days later. A total of 180 measurements was expected: 90 days x 2 measurements a day. The adherence rate was calculated as follows: number of measurements made/180 potential measurements × 100%. This ratio provides a robust measure of adherence. With this formula, adherence ranges between 0% (in case the patient did not make any measurement during her pregnancy) to over 100% (in case the pregnant woman performed more than 2 BP measurements a day). The study population of pregnant women was, in discussion with midwives and gynecologists, divided into 4 study groups: (1) Those with an adherence rate<30% (low adherence). An adherence rate below 30% is really insufficient in the follow-up of the pregnant women. When the last blood measurement of a women remains far from the clinical threshold (90 mmHg Diastolic or 140 mmHg Systolic BP), it is not critical to receive only 1 measurement in 2 days (corresponds with 30%). Whereas 30% adherence rate is way too low when the currently (and last) BPs where elevated; (2) Those with an adherence rate of 30% to 80% (moderate adherence); (3) Those with an adherence rate of 80% to 100% (good adherence); and (4) Those with an adherence rate>100% (overadherence). A large group of women seemed to be really motived to adherence to the monitoring program, and the bulk of these women seem to fall within the 30 to 80% adherence rate. In discussions with midwifes and gynecologists, women with adherence rates between 80 to 100% seemed to be a highly motivated group, whereas those with adherence rates>100% were considered to be perhaps overly anxious and concerned. An additional analysis was performed based on equal sample size groups. This analysis can be found in Multimedia Appendix 1.

### Statistical Analysis

Data were analyzed with the RStudio version 3.2.2 (RStudio Inc) statistical software. The Shapiro-Wilk test was used to assess whether the data were normally distributed. Nonparametric tests were used when the normality assumption was violated. Non-normally distributed data are expressed as medians and interquartile ranges (IQRs). Analysis of variance was used to test within-group comparisons. An independent *t* test (parametric) and/or the Mann-Whitney *U* test (nonparametric) was used for between-group comparisons. Single linear regression was performed to determine which personal characteristics had a significant relation with the adherence rate. *P* values <.05 were considered statistically significant.

## Results

### Participant Demographic and Obstetric Characteristics

In total, 108 participants completed the questionnaires. The patient demographic and obstetric characteristics are presented in Table 1. In the total sample, the median adherence was 89.4% (IQR: 54.7-103.3), the median age was 30 years (IQR: 28-33), the median prepregnancy weight was 76 kg (IQR: 66-91), the mean height was 167 cm (SD 7), the median body mass index was 27 kg/m^2^ (IQR: 24-32), and 37.9% (41/108) of the women were primiparous. There were no significant differences in any of these demographic or obstetric characteristics among the 4 adherence groups (see Table 1).

### Relationships Between Patient Characteristics, Questionnaire, and Adherence to Remote Monitoring

As expected, the results showed that several patient characteristics were associated with adherence, particularly in the groups with lower levels of adherence, although unexpected findings also emerged (see Multimedia Appendix 2). Specifically, participants in the moderate adherence group were characterized by the highest levels of anxiety and depression, particularly compared with the overadherence group, although these differences were quite modest. However, the moderate adherence group showed significantly elevated levels of rumination, magnification, and helplessness (cognitive factors) and elevated levels of both attachment anxiety and avoidance compared with the good and overadherence groups. There were no significant differences between the good and overadherence groups. Contrary to expectation, self-criticism and dependency were not associated with adherence. Other-oriented perfectionism was the only patient personality trait that distinguished the low adherence group from the other 3 groups, suggesting that this group of patients was characterized by high levels of criticism toward others, and particularly toward others who failed to meet their expectations.

Single linear regression showed that the PHQ-9 (*P*=.01) and ECR-R (*P*=.01) questionnaires were significantly related to the adherence rate. Multimedia Appendix 2 describes for each questionnaire and adherence group the median (IQR) or mean (SD) and *P* values.

**Table 1:** Characteristics of the study participants.

Variable	Low adherence, range: (0.0-27.8)	Moderate adherence, range: (36.1-78.3)	Good adherence, range: (81.7-100.0)	Overadherence, range: (100.6-156.1)	*P* value
Number of participants, n	12	32	31	33	—^a^
Adherence (%), median (IQR^b^)	9.2 (0.0-18.6)	56.4 (47.2-71.4)	90.4 (88.3-97.5)	107.2 (103.9-116.1)	—
Age (years), median (IQR)	31 (29-34)	30 (28-36)	30 (28-32)	30 (28-33)	.77
Prepregnancy weight (kg), median (IQR)	76 (64-88)	78 (68-90)	72 (67-87)	82 (65-95)	.37
Height (cm), mean (SD)	170 (4)	166 (6)	168 (8)	166 (7)	.89
BMI^c^ (kg/m^2^), median (IQR)	28 (22-32)	28 (24-31)	26 (24-30)	29 (24-34)	.36
Primigravida, n (%)	7 (58)	8 (25)	7 (23)	19 (58)	.15

^a^No significance test was performed for this descriptive demographic variable.

^b^IQR: interquartile range.

^c^BMI: body mass index.

## Discussion

### Principal Findings

We investigated the relationships between patient characteristics questionnaires of pregnant women at risk of PIH and their adherence rates in an RM program. To our knowledge, this is the first study to investigate the potential role of patient characteristics questionnaires in adherence to an RM program for pregnant women at risk of PIH. A total of 3 interesting sets of findings emerged.

First, as expected, women exhibiting moderate adherence showed higher scores for negative psychosocial traits. Specifically, the moderately adherent group was notably characterized by high levels of both attachment avoidance and anxiety as well as tendencies to ruminate, feel helpless, and magnify problems. It seems these women may have shown suboptimal adherence to the program because they worried highly and ruminated upon potential negative outcomes. As a result, they may want to avoid any potential confrontation with threatening information. If this assumption is correct, this has important implications for the future implementation of RM because the early identification of these women may increase their adherence and thus, prevent negative outcomes.

Second, although women in the low adherence group (<30% adherence) seemed to have similar psychosocial characteristics to the women who showed good and overadherence, they were distinguished by markedly elevated levels of other-oriented perfectionism. This suggests that these women display poor adherence because they are very critical of others, perhaps including the health care staff proposing and initiating RM. As other-oriented per “difficult to reach,” particularly with an intervention that involves very little personal contact between the patient and the health care provider. Therefore, when RM is implemented, it may be crucial to screen for these traits and to develop a preferably brief and cost-effective intervention to address these issues in such women.

Third, in marked contrast to our expectations, there were no significant differences between the good and overadherence groups. We expected traits such as high levels of self-criticism and rumination to be associated with very high levels of adherence, reflecting a maladaptive preoccupation with RM, leading to excessive health care behaviors. However, none of these traits distinguished this group of mothers from those with good adherence. Therefore, what we initially thought would reflect “high” adherence (ie, measuring BP more than the requested 2 times a day), might reflect the normal “maternal preoccupation” with the health of their baby that characterizes mothers in the peripartum period [28]. This maternal preoccupation is thought to reflect a biological and psychological preparedness to give birth, which from an evolutionary perspective, is adaptive. Therefore, these findings warn against interpreting the seemingly overadherence of mothers to RM as problematic. Of course, there may be a group of mothers for whom this normative preoccupation becomes a maladaptive preoccupation, but further research is required to investigate this.

Finally, the findings of this study showed a significant relation between the PHQ-9 and ECR-R questionnaire and the adherence rate.

### Strengths and Limitations

Our study is the first to demonstrate relationships between patient psychosocial characteristics and adherence rates in an RM program. Our results should contribute to an increased use of RM in obstetric care, as encouraged by the European Communities in the eHealth Action Plan. An awareness of the influence of patient characteristics on adherence rates can be useful in selecting particular pregnant women for an RM program. Although the results of this study are encouraging, a number of limitations must be taken into account in future research. First, the generalizability of the results may be affected by the single-center design of the study. Second, the results of the study relied on self-reported data. To include diagnoses in questionnaires, for example GAD-7, clinical diagnostic interviews would be required. Furthermore, the difficulty in assessing depression prenatally is that several symptoms of depression, such as fatigue, appetite change, and sleep problems, are also associated with pregnancy. During clinical diagnostic interviews, a study-specific guide can be used to determine whether the study participants perceive their symptoms to be pregnancy-related [29,30]. Third, the questionnaires were completed on a single moment basis. It is possible that exceptional events influenced the women’s responses to the questionnaires.

The overall adherence rate in this study (mean 79.56%; median 89.44%) corresponds to reported rates of 70.40% and 90.00% adherence to BP measurements [31,32]. As reported by many studies, the adherence rate usually decreases steadily over time. This reduction was more evident in the first weeks or months after the start of RM [32,33]. Participants’ nonadherence to the manual entry of daily information, especially in long-term monitoring programs, is also a problem [34].

### Recommendations for Further Research

Multiple trajectories and predictors of health-related quality of life (HRQOL) have been determined in women during pregnancy. For instance, young maternal age, low education, financial dissatisfaction, unplanned pregnancy, pregnancy-related symptoms, depression, and domestic violence may be associated with low HRQOL [35]. Future studies should investigate the influence of these variables on adherence rates to RM. The results of the low adherence group may indicate that these mothers underreport distress because of a critical and hostile attitude toward RM. Future research is required to investigate this issue. The study of Biaggi et al [36] demonstrated that depression rates tend to increase with each trimester and that anxiety and pain interference also increases significantly over time during the third trimester [37,38]. Future research should confirm the results of this study in other longitudinal periods of pregnancy. Maternal anxiety during pregnancy is associated with several adverse outcomes, including spontaneous abortion, increased cesarean section, pre-eclampsia, placental abruption, preterm labor, low birth weight, smaller head circumference, and lower mental development scores in infants [39,40]. Future research should investigate the relationships between several adverse outcomes of pregnancy and adherence rates.

### Conclusions

The peripartum period has long been known to be associated with increased levels of stress and anxiety, which can be exacerbated by PIH and negatively influence adherence rates. This study shows that anxiety, depression, and negative cognitive and attachment styles, but also other-oriented perfectionism, are characteristic of women with less than optimal adherence. As the results of the low adherence group threaten both the well-being and the follow-up of the patient, further research is required to determine possible strategies to improve the management of PIH.

## Appendix

Multimedia Appendix 1 Adherence analysis based on equal sample size groups.

Multimedia Appendix 2 Answers questionnaires related to adherence groups.

## Abbreviations

BP: blood pressure
ECR-R: Experiences in Close Relationships-Revised Scale
GAD-7: Generalized Anxiety Disorder-7
HRQOL: health-related quality of life
IQR: interquartile range
PHQ-9: Patient Health Questionnaire-9
PIH: pregnancy-induced hypertension
PREMOM: Pregnancy Remote Monitoring
RM: remote monitoring
