# Supplementary material for: Relationship Between Adherence to Remote Monitoring and Patient Characteristics: Observational Study in Women With Pregnancy-Induced Hypertension
Source: JMIR Mhealth Uhealth. 2019 Aug 28;7(8):e12574. doi: 10.2196/12574 (PMC6737887; doi:10.2196/12574)
Supplement: Multimedia Appendix 1 [file mhealth_v7i8e12574_app1.pdf]

	Low adherence<br><br>range:<br>(0.00 – 52.22)	Moderate adherence<br><br>range:<br>(55.56 – 88.89)	Good adherence<br><br>range:<br>(90.00 – 100.00)	High adherence<br><br>range:<br>(103.30 – 156.10)	P-value
Adherence (%), median [IQR]	29.67<br>(12.75-47.22)	75.56<br>(68.61-85.83)	97.79<br>(95.56-100.00)	111.10<br>(105.00-119.40)	
Age (years), median [IQR]	31.00<br>[28.00-36.00]	30.00<br>[28.00-33.]	29.00<br>[27.50-31.50]	30.00<br>[29.00-33.00]	0.408
Pre-pregnancy weight (kg), median [IQR]	78.50<br>[66.00-96.50]	71.50<br>[67.50-80.00]	74.00<br>[66.00-94.50]	80.00<br>[63.50-93.00]	0.362
Height (cm), *M* (*SD*)	168.56 ± 5.91	166.22 ± 5.72	166.78 ± 7.74	167.19 ± 7.56	0.63
BMI (kg/m^2), median [IQR]	28.37<br>[24.00-33.48]	26.08<br>[23.74-28.80]	27.73<br>[23.23-33.90]	26.77<br>[23.65-31.77]	0.465
Primigravida, *n* (%)	10 (37.04)	8 (29.63)	9 (33.33)	14 (51.85)	0.748

	Adherence groups				p						
	Low adherence	Moderate adherence	Good adherence	Over adherence	1-2	1-3	1-4	2-3	2-4	3-4	Linear regression[a]
**Anxiety and depression**											
GAD-7	4.0 [3.0-8.0]	6.0 [3.0- 8.5]	6.0 [2.5-7.5]	4.0 [2.0-5.5]	.81	.81	.14	.65	.07	.19	-.15
PHQ-9	5.0 [3.0-10.5]	4.0 [2.5-6.5]	4.0 [2.0-6.0]	2.0 [2.0-5.5]	.36	.08	.02*	.32	.08	.53	-.25*
**Cognitive factors**											
PCS											
rumination	7.0 [3.0-11.5]	6.0 [2.0-9.0]	5.0 [2.0-9.0]	4.0 [2.0-8.5]	.24	.10	.04*	.69	.49	.66	-.14
magnification	5.0 [2.0-8.0]	4.0 [3.0-6.5]	4.0 [3.0-6.5]	4.0 [2.5-5.0]	.64	.51	.21	.85	.47	.58	-.12
helplessness	7.0 [4.0-12.5]	7.0 [3.5-9.5]	6.0 [3.0-11.0]	5.0 [3.5-8.0]	.48	.32	.14	.79	.34	.14	-.13
**Attachment and personality**											
ECR-R											
avoidance	2.9 [2.4-3.8]	2.6 [2.2-2.9]	2.5 [1.8-2.8]	2.3 [1.8-2.9]	.10	.03*	.02*	.56	.42	.02*	-.27*
anxiety	3.1 [2.7-3.9]	3.0 [2.4-3.1]	2.8 [2.5-3.1]	2.7 [2.3-3.1]	.19	.06	.02*	.86	.31	.40	-.24*

DEQ-A											
dependency	35.5 ±9.7	33.0±8.3	32.3±7.3	33.6±6.7	.32	.17	.40	.73	.79	.50	-.17
self-criticism	27.0 [23.5-30.5]	26.0 [22.0-30.0]	25.0 [22.0-29.5]	26.0 [23.5-30.5]	.51	.48	.72	.95	.76	.80	-.14
MPS											
self-oriented perfectionism	62.6 ±17.2	58.1±16.7	67.4±15.3	62.7±17.4	.35	.40	.95	.08	.34	.36	-.02
other oriented perfectionism	51.0±11.4	46.5±9.2	51.3±8.7	46.8±7.4	.08	.70	.07	.10	.99	.10	-.16
Socially prescribed perfecti	48.0 ±10.1	47.4±11.7	46.7±10.4	47.2±12.8	.74	.87	.85	.90	.86	.94	-.07

Note: 1, Low adherence group; 2, Moderate adherence group; 3, Good adherence group; 4, High adherence group;

[a] Beta-value of the covariate of the single linear regression model with the compliance rate as the dependent variable and only one specific questionnaire included as a covariate

*significant at $P < 0.05$
