# Supplementary material for: Relationship Between Adherence to Remote Monitoring and Patient Characteristics: Observational Study in Women With Pregnancy-Induced Hypertension
Source: JMIR Mhealth Uhealth. 2019 Aug 28;7(8):e12574. doi: 10.2196/12574 (PMC6737887; doi:10.2196/12574)
Supplement: Multimedia Appendix 2 [file mhealth_v7i8e12574_app2.pdf]

Table 2. Answers questionnaires related to adherence groups. Data are expressed as median (IQR) or mean (SD).

	Adherence groups				p						
	Low adherence	Moderate adherence	Good adherence	Over adherence	1-2	1-3	1-4	2-3	2-4	3-4	Linear regression[a]
**Anxiety and depression**											
GAD-7	4.0 [2.8-6.8]	6.0 [3.0- 8.3]	6.0 [2.0-8.0]	4.0 [2.0-6.0]	.40	.62	.61	.55	.00*	.14	-.15
PHQ-9	3.5 [2.8-8.0]	5.0 [2.8-8.3]	4.0 [2.0-7.0]	3.0 [2.0-5.0]	.78	.62	.21	.27	.03*	.29	-.25*
**Cognitive factors**											
PCS											
rumination	4.5 [1.0-7.5]	7.0 [4.0-12.0]	5.0 [2.0-9.5]	4.0 [2.0-8.0]	.07	.50	.97	.07	.01*	.32	-.14
magnification	3.0 [2.0-6.0]	6.0 [3.0-8.0]	4.0 [2.5-7.0]	4.0 [3.0-5.0]	.07	.48	.77	.20	.03*	.62	-.12
helplessness	5.0 [2.8-8.0]	7.5 [5.5-12.3]	6.0 [3.0-12.0]	5.0 [3.0-8.0]	.07	.49	.79	.19	.02*	.47	-.13
**Attachment and personality**											
ECR-R											
avoidance	2.6 [2.2-3.4]	2.8 [2.3-3.5]	2.6 [2.1-2.9]	2.3 [1.8-2.9]	.98	.37	.19	.22	.049*	.56	-.27*
anxiety	2.8 [2.6-3.5]	3.0 [2.7-3.6]	2.8 [2.5-3.2]	2.7 [2.3-3.1]	.66	.66	.32	.17	.042*	.31	-.24*

DEQ-A											
dependency	36.0 ±7.8	34.5±10.0	32.0±7.6	33.4±6.2	.60	.14	.32	.27	.63	.40	-.17
self-criticism	27.0<br>[24.0-29.8]	28.0<br>[23.0-30.3]	26.0<br>[21.0-29.0]	25.0<br>[23.0-30.0]	.80	.38	.47	.45	.69	.79	-.14
MPS											
self-oriented perfectionism	71.0 ±11.4	56.8±17.6	65.7±15.8	62.6±17.0	.00*	.24	.07	.04	.18	.45	-.02
other oriented perfectionism	57.1±3.9	45.5±10.6	50.1±8.6	48.1±8.7	.00*	.00*	.00*	.06	.28	.35	-.16
Socially prescribed perfecti	50.0 ±11.4	46.4±8.9	47.6±12.3	47.1±12.2	.34	.54	.46	.68	.81	.87	-.07

Note: 1, group with less than 30% adherence; 2, group with 30%–80% adherence; 3, group with 80%–100% adherence; 4, group with more than 100% adherence;

[a] Beta-value of the covariate of the single linear regression model with the compliance rate as the dependent variable and only one specific questionnaire included as a covariate

*significant at P < 0.05
